# Membrane-bound exosomal HSP70 as a biomarker for detection and monitoring of malignant solid tumours: a pilot study

**DOI:** 10.1186/s40814-020-00577-2

**Published:** 2020-03-03

**Authors:** Gaétan Chanteloup, Marine Cordonnier, Nicolas Isambert, Aurélie Bertaut, Guillaume Marcion, Carmen Garrido, Jessica Gobbo

**Affiliations:** 1 Laboratoire d’Excellence LipSTIC, UMR 1231, INSERM, Dijon, France; 2 Faculty of Medicine and Pharmacy, University of Burgundy, Dijon, France; 3 Pôle Régional de Cancérologie, CHU de Poitiers Poitiers, INSERM U-1084, University de Poitiers, Poitiers, France; 4 Unit of Methodology, Biostatistics and Data Management, Georges-François Leclerc Centre, Dijon, France; 5 CIC-1432, INSERM, Dijon, France; 6 Department of Medical Oncology, Early Phase Unit, Georges-François Leclerc Centre, 1, Rue du Professeur Marion, 21079 Dijon, France

**Keywords:** Pilot study, Liquid biopsy, HSP70-exosomes, Solid tumours, Cancer diagnosis and monitoring

## Abstract

**Background:**

Cancer is the second leading cause of death globally. Early detection and disease management lead to a better survival rate. Consequently, discovery of novel methods in cancer early diagnosis is a field of active research. Minimally invasive liquid biopsies are generating growing interest. Circulating tumour cells (CTCs) have been identified in patients’ blood; nevertheless, these cells are rare and heterogeneous. Exosomes are extracellular nanovesicles released into the extracellular environment via the endosomal vesicle pathway and found in different body fluids. Exosomes deliver bioactive cargo such as proteins, mRNA and miRNA to recipient cells in the tumour environment. We have recently shown that heat shock protein 70 (HSP70) is detected in the membrane of tumour-derived exosomes, in contrast to normal cells. One single cancer cell can release thousands of HSP70-exosomes, facilitating detection. The aim of the pilot study ExoDiag is to determine whether it is possible to detect and quantify HSP70-exosomes in blood in patients with solid cancers.

**Methods:**

Bicentric pilot study that will include 60 adult patients with metastatic and non-metastatic solid tumours and 20 healthy volunteers. Exosomes will be isolated from blood and urine samples, and HSP70 concentration will be determined. Patients will be followed for 1 year. The study is sponsored by Georges-François Leclerc Centre and is currently ongoing.

**Discussion:**

We expect to demonstrate that HSP70-exosomes could be a powerful tool to diagnose cancer and to guide clinicians in therapeutic decision-making, improving patient’s care.

**Trial Registration:**

ClinicalTrials.gov identifier NCT02662621. Registered 20 January 2016, https://clinicaltrials.gov/ct2/show/study/NCT02662621?term=NCT02662621&rank=1

## Background

Cancer is one of the most lethal diseases due to lack of early diagnosis. In 2018, the global cancer burden is estimated to 18.1 million new cancer cases and 9.6 million cancer deaths worldwide, according to the International Agency for Research on Cancer (IARC) [1]. In France, prostate and breast cancer are the most common cancers among men and women, respectively. Lung, colon, and breast cancer represent more than one third of cancer-related deaths [1]. In 20 years, cancer death rate decreased 22% in men and 14% in women (INCa). This decrease is correlated not only to treatment improvement but also to earlier diagnosis. It is well known that development of efficient diagnosis methods is crucial to reduce the cancer-related morbidity.

Current solid biopsy methods of tumour tissues are critical for the diagnosis and molecular testing of cancer. Nevertheless, several limitations have been observed in current sampling methods such as invasiveness, recovery of a limited amount of biomaterials and difficulties in repeated sampling. Moreover, tumour heterogeneity may render difficult representability of a particular tumour tissue biopsy [2, 3]. Therefore, liquid biopsies, collecting body fluids such as blood, urine, effusions or saliva, through a minimally invasive method are currently tested to identify biomarkers relevant for cancer diagnosis and dynamic monitoring.

Circulating tumour cells (CTCs) have been identified in blood. These cells are extremely rare, 1 CTC to 10^6^–10^7^ leukocytes, in particular in early stage disease [4]. A test to detect CTCs of epithelial origin has been approved by FDA [5]; nevertheless, this technique has not been implemented into routine clinical practice [6]. Indeed, epithelial cell adhesion molecules used to detect CTCs are not present at the surface of all CTCs.

Exosomes are nanovesicles (diameter 50–200 nm) secreted by numerous cell types and derived from the endosomal pathway. These extracellular nanovesicles form a bioactive cargo retrieved in several body fluids including the urine [7] and blood [8].These vesicles are composed of a cholesterol-enriched membrane enclosing proteins and genetic material. It has been shown that tumour-derived exosomes play an important role in cancer development [9]. We have shown that heat shock protein 70 is detected in the membrane of tumour-derived exosomes, in contrast to untransformed cells [10]. HSPs, also called stress proteins, are highly conserved molecular chaperones that participate in protein folding, activity, transport and stability [11, 12]. Among the different HSPs, HSP70 is induced by different types of stress and exhibits cell protective properties through its interaction with key factors in cell death pathways. HSP70 has an anti-apoptotic function, and its overexpression in cancer cells [13] confers resistance to chemotherapeutic drugs, thereby promoting cancer development [14]. Therefore, HSP70 expression level may predict chemotherapy response [15]. Indeed, HSP70 expression correlates with poor prognosis in breast, prostate, endometrial, uterine, cervical and bladder carcinomas [16]. Interestingly, HSP70 can be released in the extracellular space acting like danger signal [17]. Moreover, this protein can be expressed in a membrane-bound form, exposing in the extracellular media a 14 amino-acid C-terminal loop. We have demonstrated that HSP70 can be secreted via exosomes. In contrast to normal cells/untransformed cells, overexpression of HSP70 in cancer cells leads to its membrane translocation and its consequent detection in exosome surface. We have named these nanovesicles HSP70-exosomes [10, 18]. Their involvement in tumour growth through inhibition of the immune response and enhancement of metastatic niche formation has been demonstrated [14, 19]. These data prompted us to test whether HSP70-exosomes could be used as a cancer biomarker. Detection of HSP70-exosomes in the urine and blood of cancer patients could overcome limitations observed with CTCs since (i) exosomes are found in large amounts compared to CTCs (e.g. 53.2 ± 1.6 × 10^8^ exosomes per 10^6^ cells in the 24 h period, determined by Nanoparticle Tracking Analysis [20], (ii) HSP70 is broadly expressed in cancer cells and its overexpression in these cells leads to its membrane translocation and consequent detection in the exosomal membrane. (iii) We have patented an interference biolayer (BLI) protocol to easily capture HSP70-exosomes isolated from human fluids using as a high affinity ligand our peptide aptamer A8 (WO2015/189395, Inserm transfer, 2014).

Multiple clinical studies are currently ongoing to investigate the use of exosomes as a diagnosis tools in the context of specific cancer subsets. In this study, we will test whether detection and quantification of HSP70-exosomes in a liquid biopsy of patients suffering from different types of solid tumours could be used to cancer early detection, to predict response to treatment and to allow disease monitoring. This approach could provide a novel, non-invasive, sensitive and fast method in diagnosis and dynamic monitoring of cancer.

## Methods

### Study population

**Fig. 1:**
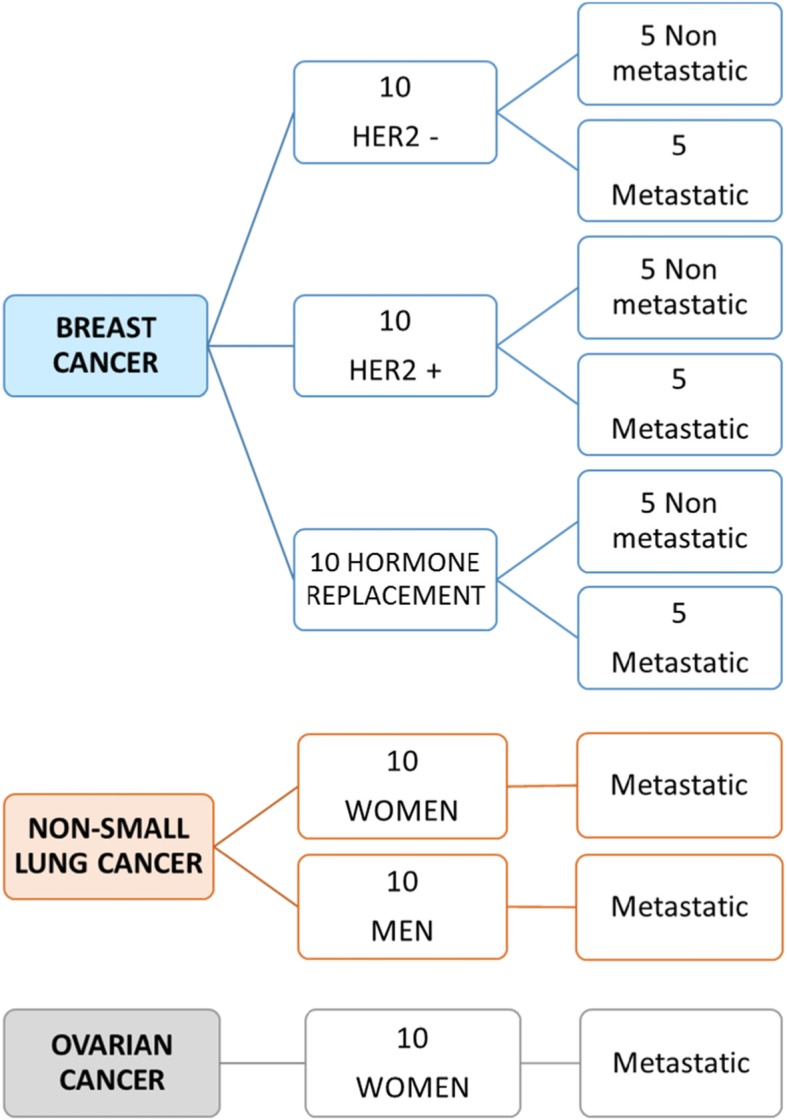
Study population

Sixty adult patients with either non-metastatic or metastatic solid tumours will be included in the study. Briefly, 30 women with breast cancer whose, (i) 10 HER2-positive (5 non-metastatic and 5 with a first metastatic evolution), (ii) 10 HER2-negative (5 non-metastatic and 5 with a first metastatic evolution), (iii) 10 on hormone replacement therapy (5 non-metastatic and 5 with a first metastatic evolution), 10 women with an ovarian cancer grade III and IV, 10 men and 10 women with a metastatic non-small cell lung cancer and 20 healthy donors will be included in the study (Fig. 1). Eligibility criteria for cancer patients are listed in Table 1. Additionally, 20 healthy volunteers with no previous cancer history, negative serology for HIV, HCV and HBC and aged 50 to 70 years old will also be included. Age group of healthy donors is in agreement with the average age of occurrence of the different solid tumours studied.

**Table 1:** EXODIAG study inclusion and exclusion criteria for cancer patients

Inclusion criteria for cancer patients	
Women newly diagnosed with infiltrating non-metastatic breast cancer (positive or negative HER status or hormone therapy). Women with breast cancer with a first metastatic evolution (positive or negative HER status or hormone therapy).	
Women newly diagnosed with stage III and ICV ovarian cancer. Men and women newly diagnosed with metastatic non-small cell lung cancer.	
Patients older than 18 years. Performance status of 0 or 1 at the time of inclusion, according to the Eastern Cooperative Oncology Group (ECOG) and WHO.	
Affiliated to the French social security social or beneficiary of such a regimen.	
Written informed consent.	
Exclusion criteria for cancer patients	
Patient with another synchronous tumour.	
Men with breast cancer.	
Positive serology for HIV, HCV or HBV.	
Patients unable to undergo medical follow-up for geographical, social or psychological reasons.	
Pregnant or nursing women.	

### Study objectives and endpoints

The primary objective of this study is to determine whether it is possible to detect and quantify HSP70-exosomes in blood in patients with solid cancers.

The secondary objectives are to:

1. determine whether the amount of HSP70-exosomes can be associated to:

(i) response to treatment;
(ii) metastatic evolution;

2. determine whether the concentration of HSP70-exosomes varies with the nature of the primary tumour or the effectiveness of the treatment used;
3. compare the concentrations and the early detection in HSP70-exosomes and those in circulating tumour cells determined by CellSearch, at inclusion and during patient follow-up;

The primary endpoint of the study is the blood concentration of HSP70-exosomes in malignant solid tumour patients and cancer-free controls. The secondary endpoints are as follows:

(i) the concentration of blood HSP70-exosomes in patients having a complete response, partial response, stable disease or progressive disease (RECIST criteria);
(ii) the concentration of blood HSP70-exosomes in patients having metastases versus non-metastatic patients;

This study includes optional ancillary studies whose aims are to detect and quantify HSP70-exosomes in patients’ urine and to determine the genetic signature of miRNAs in HSP70-exosomes isolated from patients’ blood and urine and whether these miRNAs can be predictive of treatment response.

### Study design

**Fig. 2:**
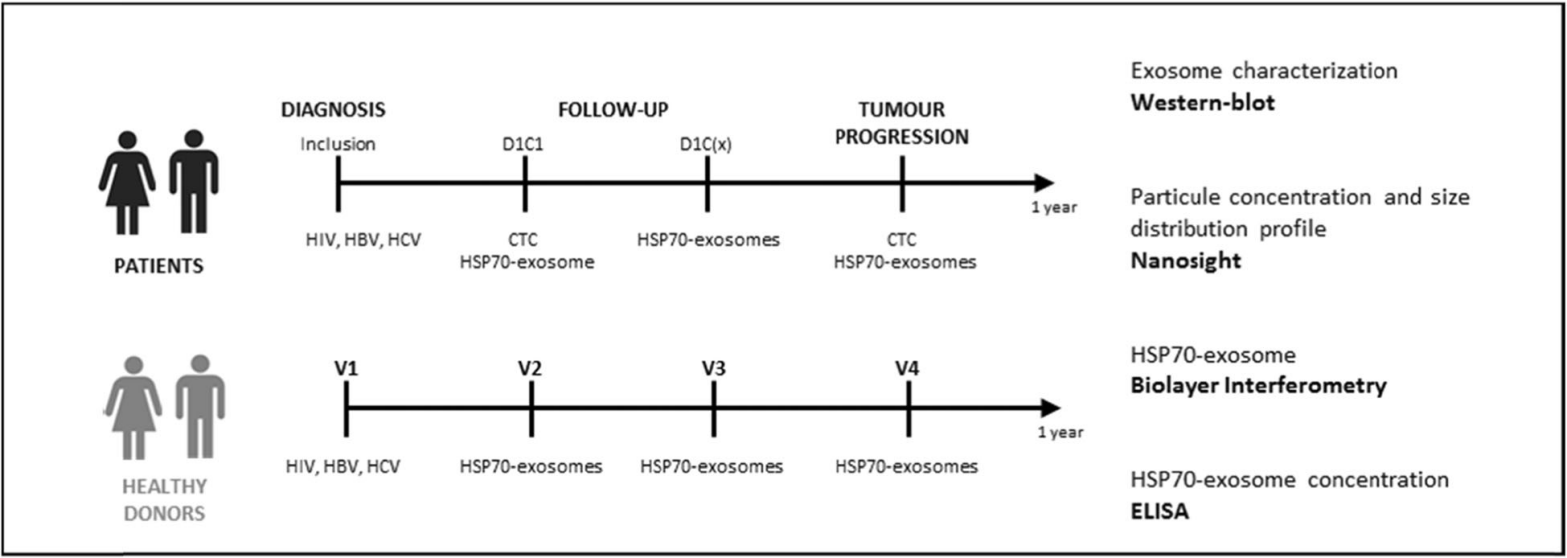
Study design

Investigator will provide eligible patients with an informative notice on ExoDiag study. Upon patient written consent, serology to HIV, HCV and HBV will be tested (Fig. 2). Only patients with negative serology for these infectious agents will be included, to protect technicians from biological risks, in agreement with laboratory procedures. During the study, 10 ml of blood, collected in an EDTA tube, will be necessary for each exosomal HSP70 analysis, and additional 10 ml of blood, collected with preservative cell save (Cell Search®) will be required for each CTCs analysis. Study design is depicted in Fig. 2.

#### Initial sampling

Prior to any treatment (radiotherapy, hormone therapy, surgery or chemotherapy) blood will be collected for exosomal HSP70 and CTCs analysis (Fig. 2; patients D1C1, Day1Cure1).

#### Follow-up

Sample collection schedule will depend on treatment standard of care (Fig. 2; patients follow-up D1C(x), Day1CureX):

- Upon a surgical treatment, initial follow-up blood sample for HSP70 analysis will be collected during the planned post-surgery visit to the investigator site;
- Upon hormone therapy, blood sample for HSP70 analysis will be collected at each follow-up oncology visit, in average every 6 months;
- Upon chemotherapy, blood sample for HSP70 analysis will be collected every two cures (C2, C4, C6, C8); sample schedule will be maintained even if toxicity leads to treatment changes.

#### Disease progression

If disease progression is observed, blood will be collected for exosomal HSP70 and CTCs analysis (Fig. 2, patients-tumour progression).

#### Healthy donors

Blood samples will be collected every 3 months during 1 year (V1 to V4) for exosomal HSP70 analysis in healthy donors (Fig. 2, healthy donors). Moreover, in the event of cancer, healthy donors will be removed from the analysis and replaced.

#### Ancillary studies

Patients and healthy subjects will be informed on ancillary studies. Such studies will not require additional visits. If participant agrees to participate, 20 ml of first-morning specimen urine will be collected for exosomal HSP70 analysis, and additional 22.5 ml of blood (5 EDTA tube 4.5 ml) will be collected to study miRNA content of HSP70-exosomes.

### Sample analysis

Extracellular vesicles will be isolated by ultracentrifugation and characterised using a standardised protocol. Particle concentration and distribution profile will be assessed using Nanoparticles Tracking Analysis (Nanosight NS300). Expression of specific exosomal markers will be analysed by western blot. HSP70-exosomes will be captured by Biolayer Interferometry using the A8 aptamer (WO2015/189395, Inserm transfer, 2014), and HSP70 concentration will be quantified by ELISA.

### Data collection

Data collected during the study will be recorded on a CRF for patients and healthy donors. On initial visit, age, sex, weight, height, medical history and special medication will be noted. In addition, healthy donors will be followed for a year and any change in treatments or intercurrent illness. Concerning patients, upon diagnosis and during medical follow-up, date of disease diagnosis, line of treatment, date and relapse type, metastasis/metastatic sites, surgical procedures, treatment modification, date of medical exams performed during assessments (radiographic, biological) and date of death will be noted.

### Sample size and statistical analysis

A selected group of 60 patients, including 30 women with a breast cancer whose (i) 10 HER2-positive (5 non-metastatic and 5 with a first metastatic evolution), (ii) 10 HER2-negative (5 non-metastatic and 5 with a first metastatic evolution), (iii) 10 on hormone replacement therapy (5 non-metastatic and 5 with a first metastatic evolution), 10 women with an ovarian cancer grade III and IV, 10 men and 10 women with a metastatic non-small cell lung cancer and 20 healthy donors will be included in the study (Fig. 1). In this project, the main objective is to evaluate the feasibility of HSP70-exosomes dosing in patients with cancer: The assumptions related to baseline assays are as follows: a technically feasible dosage rate of 75% is considered to be insufficient, a 90% feasibility rate is expected, unilateral alpha = 5%, power = 90%, 10% rate of withdrawal of patient consent or study discontinuation before initial dosing. Based on these assumptions, using a one-step Fleming design, 60 subjects are required (55 + 10% = 60). At the end of the study on the first 55 patients included, if 47 or more patients had a dosage, the technique will be considered as having good feasibility.

In addition, baseline assays of the 60 patients will be compared with 20 controls, to confirm that exosome HSP70 is as a cancer specific marker. The mean of HSP70 in the controls is expected to be 0. It is expected to be around 2 for cancer patients with a common standard deviation of 2. With these hypotheses, bilateral alpha of 5% and 60 patients, we will have a power of more than 95% to compare exosome HSP70 concentrations between cases and controls.

Subgroup analyses, depending on the pathology, histological type and response to treatment, will be exclusively exploratory. These analyses will be carried to study whether variations in HSP70-exosome dosage at baseline and follow-up could be associated with these different variables.

In exploratory analysis, association between HSP70-exosomes concentration and treatment response (response and stable versus progressive disease) will be modelled using logistic regression and ROC curves. Area under the ROC curve will be determined with its 95% confidence interval. The same strategy will be applied to determine link between HSP70-exosomes concentration and the presence of metastases. Analyses will be repeated on subpopulation according to location of the cancer. Analyses will be performed using SAS 9.4.

### Patient and public involvement

The patient committee of the National League Against Cancer (“Comité de patients en recherche clinique en cancérologie de la Ligue contre le cancer”) was consulted on the information notice that will be given to patients asked to participate in this trial. This committee brings together patients, former patients or patient relatives who have a shared interest in oncology medical research. The purpose of this consultation was to read and improve the patient information notice on the design and aim of the study, so that patients can clearly understand and decide whether to participate, in agreement with the principle of informed consent.

## Discussion

The discovery of powerful biomarkers in cancer diagnosis and monitoring appears as a major challenge to improve patient’s care. Current research focuses on liquid biopsies that enable non-invasive detection of tumours, a clear advantage over tissues biopsies that have inherent risks. Blood samples are particularly interesting since this tissue contains cancer specific genetic material, proteins and extracellular vesicles. Tumour-derived exosomes emerge as a novel tool in cancer diagnosis and monitoring. However, most studies are focused on specific cancer subtypes, and no common marker is currently used, except CTCs. We have shown recently that human tumour cells, in contrast to their normal counterparts, release exosomes containing membrane-bound HSP70 [10]. Thus, we suggest that HSP70-exosomes could be used as a biomarker in the diagnosis and monitoring of most of cancers. For this purpose, we designed a pilot study, named ExoDiag, to demonstrate whether it is possible to detect and quantify HSP70-exosomes in blood in patients with solid cancers. We expected that HSP70-exosomes are highly secreted in the blood of patients suffering from different solid tumours (breast, ovarian and non-small cell lung cancer) when compared to healthy donors. A threshold corresponding to cancer detection will be determined. We will also investigate if the level of circulating HSP70-exosomes could be used for patient’s stratification. Furthermore, we will determine whether the level of circulating HSP70-exosomes changes upon treatment. Our aim is to establish whether HSP70-exosomes concentration is correlated to tumour regression or disease progression, thereby assessing their prognostic value in solid tumours. In long term, we hope to demonstrate that HSP70-exosomes could be a powerful tool to diagnose cancer and to guide clinicians in therapeutic decision-making, improving patient’s care.

To our knowledge, this will be the first study to evaluate the technical feasibility of measuring HSP70 in exosomes in cancer patients. The results of this pilot study might highlight the strong potential of liquid biopsy through exosomes in the monitoring disease progression. Therefore, if this pilot study is successful, an international multicentre study will be envisaged.

Main study limitation might be the exosome isolation technical procedure, difficult to translate to a routine clinical setting, notably due to the ultracentrifugation step. Lack of standardisation in the exosome field is an important break for its clinical use. Nevertheless, as technological advances are fast, we are confident that new procedures will soon enable exosome analysis using a standardised method.

## Data Availability

This section is not applicable.

## Abbreviations

CTC: Circulating tumour cells
ECOG: Eastern Cooperative Oncology Group
HBV: Hepatitis B virus
HCV: Hepatitis C virus
HER2: Human epidermal growth factor receptor 2
HIV: Human immunodeficiency virus
HSP70: Heat shock protein 70 kDa
miRNA: Micro ribonucleic acid
WHO: World Health Organization
